# Cell surface heparan sulfate proteoglycans contribute to intracellular lipid accumulation in adipocytes

**DOI:** 10.1186/1476-511X-4-2

**Published:** 2005-01-06

**Authors:** Larissa C Wilsie, Shree Chanchani, Deepti Navaratna, Robert A Orlando

**Affiliations:** 1 Department of Biochemistry and Molecular Biology, University of New Mexico, Health Sciences Center, 915 Camino de Salud, Albuquerque, New Mexico, 87131, USA

## Abstract

**Background:**

Transport of fatty acids within the cytosol of adipocytes and their subsequent assimilation into lipid droplets has been thoroughly investigated; however, the mechanism by which fatty acids are transported across the plasma membrane from the extracellular environment remains unclear. Since triacylglycerol-rich lipoproteins represent an abundant source of fatty acids for adipocyte utilization, we have investigated the expression levels of cell surface lipoprotein receptors and their functional contributions toward intracellular lipid accumulation; these include very low density lipoprotein receptor (VLDL-R), low density lipoprotein receptor-related protein (LRP), and heparan sulfate proteoglycans (HSPG).

**Results:**

We found that expression of these three lipoprotein receptors increased 5-fold, 2-fold, and 2.5-fold, respectively, during adipocyte differentiation. The major proteoglycans expressed by mature adipocytes are of high molecular weight (>500 kD) and contain both heparan and chondroitin sulfate moieties. Using ligand binding antagonists, we observed that HSPG, rather than VLDL-R or LRP, play a primary role in the uptake of DiI-lableled apoE-VLDL by mature adipocytes. In addition, inhibitors of HSPG maturation resulted in a significant reduction (>85%) in intracellular lipid accumulation.

**Conclusions:**

These results suggest that cell surface HSPG is required for fatty acid transport across the plasma membrane of adipocytes.

## Background

The adipocyte plays a central role in overall metabolic regulation serving as a storage depot for fatty acids and as an endocrine cell to regulate energy utilization and feeding behavior [1,2]. The mass of adipose tissue is maintained by a well-controlled balance of cell proliferation (hyperplasia) and increase in fat cell size (hypertrophy). Contributing to adipocyte hypertrophy is the assimilation of fatty acids into cytosolic triacylglycerol-rich lipid droplets. Fatty acids enter the adipocyte through the plasma membrane, are converted to their acyl-CoA derivatives and transported through the cytosol with the assistance of fatty acid binding proteins due to the lipophilic nature of the fatty acid hydrocarbon chain [3,4]. They are then reassembled into triacylglycerol units by acyltransferases. The intracellular lipid droplet that forms from the coalescence of triacylglycerols has recently been shown to associate with regulators of membrane trafficking in addition to enzymes needed for fatty acid storage and utilization, suggesting a complex and dynamic role deserving of the name adiposome [5].

Extracellular fatty acids that are available for adipocyte uptake are either 1) associated with circulating albumin, 2) hydrolyzed from triacylglycerol-rich lipoprotein particles by lipoprotein lipase, or 3) in the form of VLDL particles which can be directly internalized by adipocyte lipoprotein receptors. In the circulation, VLDL represents the major source of fatty acids for peripheral tissues in the form of triacylglycerols and provides a concentrated source of esterified fatty acids. It is interesting that in light of the well studied processes of cytosolic transport and assimilation of free fatty acids into triacylglycerol-rich storage droplets, the mechanism of transport of fatty acids across the adipocyte plasma membrane remains controversial. Two mechanisms, which are not mutually exclusive, have been proposed: one involves passive diffusion across the plasma membrane [6,7], the other requires protein-mediated transport [8,9]. Passive diffusion, which requires protonation of the fatty acid prior to entering the bilayer, has long been regarded as the major pathway for uptake of fatty acids by cells. However, recent kinetic data suggest that passive diffusion, while sufficient for cells with relatively low metabolic rates, is likely to be insufficient for cells with high fatty acid utilization such as skeletal muscle and adipose [10-12]. Moreover, the role of fatty acid-albumin complexes as a significant source of diffusible free fatty acids has recently been questioned, as evidence indicates that a significant transfer of fatty acids from albumin occurs only at very high and non-physiological fatty acid to albumin ratios [13,14]. Protein-mediated transport of fatty acids has been investigated using fatty acid binding and uptake studies [15,16]. These results show that fatty acid permeation demonstrates concentration-dependent, nonlinear saturation kinetics with a Km of transport of ~7 nM [9]. Moreover, uptake of long-chain fatty acids (>18 carbons) was competable [17,18], further suggesting a receptor-mediated process.

Several cell surface proteins are expressed by adipocytes which potentially contribute to receptor-mediated uptake of extracellular fatty acids; these include CD36, fatty acid transport protein-1 (FATP1), very low density lipoprotein receptor (VLDL-R), low density lipoprotein receptor-related protein (LRP), and heparan sulfate proteoglycans (HSPG). CD36 is a cell surface glycoprotein that binds long-chain fatty acids with high affinity and demonstrates a subcellular distribution that is consistent with a role in fatty acid transport across the plasma membrane [19-23]. Following the induction of pre-adipocyte 3T3-L1 cells, CD36 expression increases [24] with a concomitant increase in fatty acid uptake. *In vivo *studies corroborate these observations as uptake of long-chain fatty acids is impaired in CD36 knock-out mice [25] or when CD36 expression is reduced by anti-sense RNA treatment [22]. Although these data indicate a significant role for CD36 in long-chain fatty acid uptake, the data further suggest that it is not sufficient to account for the entirety of fatty acid uptake by adipocytes since the absence of CD36 reduces uptake by only 50% [25]. FATP1 is a 71 kD transmembrane protein and the major FATP family member expressed in adipocytes [26]. In adipocytes, insulin is known to induce the translocation of FATP1 from a perinuclear compartment to the plasma membrane [27]. Similar to CD36, FATP1 expression enhances the uptake of fatty acids [26,28].

Studies using a VLDL-R knock-out animal model have suggested a role for this receptor in the accumulation of fatty acids by adipose tissue; mice lacking VLDL-R demonstrate only modest weight gain and a reduction in adipose tissue mass when placed on a high-fat diet [29]. VLDL-R mRNA is known to increase ~3–5-fold during adipocyte differentiation [30], suggesting that it may play a critical role in adipocyte physiology. The LRP is also expressed by adipocytes [31-33] and like VLDL-R can bind and internalize apoE-enriched VLDL particles [34,35]. However, its role in lipid accumulation by adipocytes has not been investigated. HSPG have been well characterized in their ability to bind and internalize apoE-enriched VLDL [36-38], as well as localize lipases to the cell surface through high affinity binding [39,40]. HSPG have also been shown to play a significant role in the hepatic clearance of lipoprotein, however, like LRP, no studies to date have investigated their role in intracellular lipid accumulation by adipocytes.

To better understand the process of fatty acid transport across the plasma membrane of adipocytes, we have examined the expression levels and functional contributions of lipoprotein receptors, VLDL-R, LRP and HSPG toward intracellular lipid accumulation. Our findings suggest that cell surface HSPG play an essential role in fatty acid uptake and intracellular lipid accumulation in adipocytes.

## Results

### VLDL-R and LRP protein expression increases during adipocyte differentiation

**Figure 1:**
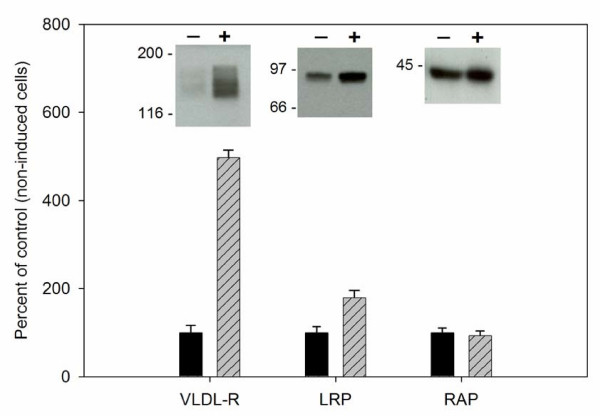
**Expression of VLDL-R and LRP increases following adipocyte differentiation. **Pre-adipocyte 3T3-L1 cells were incubated for 4 d in the presence (+) or absence (-) of 100 ng/ml dexamethasone, 100 μg/ml 3-isobutyl-1-methylxanthine, and 1 μg/ml insulin, followed by 1 μg/ml insulin for an additional 4–8 d. Total cellular protein was obtained by detergent extraction, equal amounts of protein (20 μg/lane) were separated by SDS-PAGE and immunoblotted with anti-VLDL-R polyclonal IgG (4 μg/ml), or anti-LRP (1:2000) or anti-RAP (1:2000) antisera. The chemiluminescence image was quantitated by densitometry. Values obtained for non-treated cells were assigned as 100% for comparison with treated cells. Data shown is representative of 3 separate experiments.

Since members of the LDL receptor family are known to play a predominant role in the uptake of lipoproteins, we chose to examine the protein expression levels of VLDL-R and LRP during adipocyte differentiation using 3T3-L1 cells [41], which are a well-established *in vitro *model for adipocyte differentiation [42,43]. 3T3-L1 cells were incubated with or without differentiation agents (dexamethasone, 3-isobutyl-1-methylxanthine, and insulin) and total protein extracts were immunoblotted with antibodies specific for VLDL-R, LRP, or the LDL receptor family-specific chaperone, receptor associated protein (RAP) (Fig. 1). Densitometric analysis of the resulting immunoblots indicated that VLDL-R expression increased by ~5-fold over non-differentiated, control cells. LRP expression demonstrated a more modest increase of ~2-fold over control cells. RAP showed no difference in expression between treated and non-treated cells, thus serving as a useful internal loading control. These results are consistent with previous studies which have shown an increase in mRNA levels during adipocyte differentiation for VLDL-R (3–5-fold) [30] and LRP (1.5 to 2-fold) [31].

**Figure 2:**
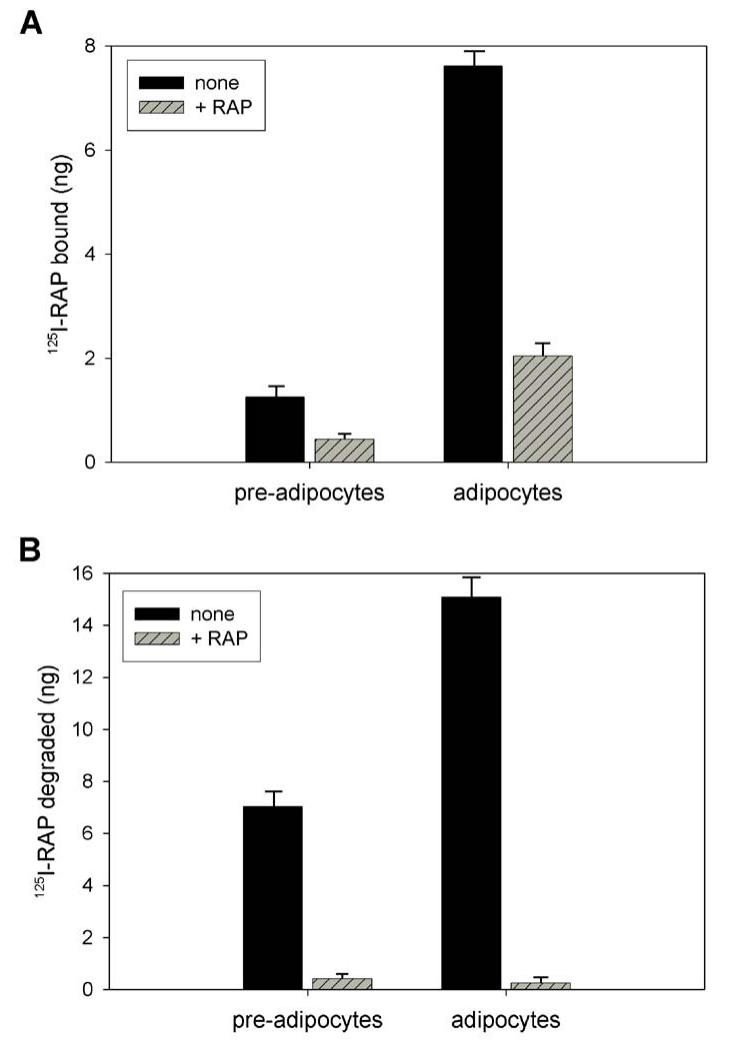
**Differentiated adipocytes bind and internalize more RAP than pre-adipocytes. **Pre-adipocyte 3T3-L1 cells and differentiated adipocytes were incubated with ^125^I-RAP (500 ng/ml) in the presence (hatched bars) or absence (solid bars) of unlabeled RAP (20 μg/ml) at 4°C for 3 h (A) or 37°C for 3 h (B). For 4°C incubations, bound ligand was solubilized and directly quantitated by scintillation counting. For 37°C incubations, culture media was processed for trichloroacetic acid (TCA) precipitation and soluble radioactivity, representing degraded ligand, was quantitated by scintillation counting.

RAP, besides being an exocytic chaperone for members of the LDL receptor family, is also a high affinity ligand for most, if not all, members of the family [44,45]. This property is useful for evaluating specific receptor binding and internalization activities by cells. Cell surface expression of LDL receptor family members was compared between control, non-treated 3T3-L1 cells and differentiated cells by incubating with ^125^I-RAP at 4°C (Fig. 2A). Differentiated cells were found to bind ~7-fold more ^125^I-RAP than non-treated control cells. This increase in receptor expression, as measured by ligand binding, is quantitatively consistent with that measured by immunoblotting. When ^125^I-RAP was incubated with cells at 37°C to evaluate receptor internalization, we found that differentiated adipocytes internalized ~2-fold more ^125^I-RAP than control 3T3-L1 cells (Fig. 2B). Together, these results indicate that levels of both VLDL-R and LRP are increased at the cell surface resulting in increased receptor internalization activity in differentiated adipocytes.

### Sulfated proteoglycan levels are increased in differentiated adipocytes

**Figure 3:**
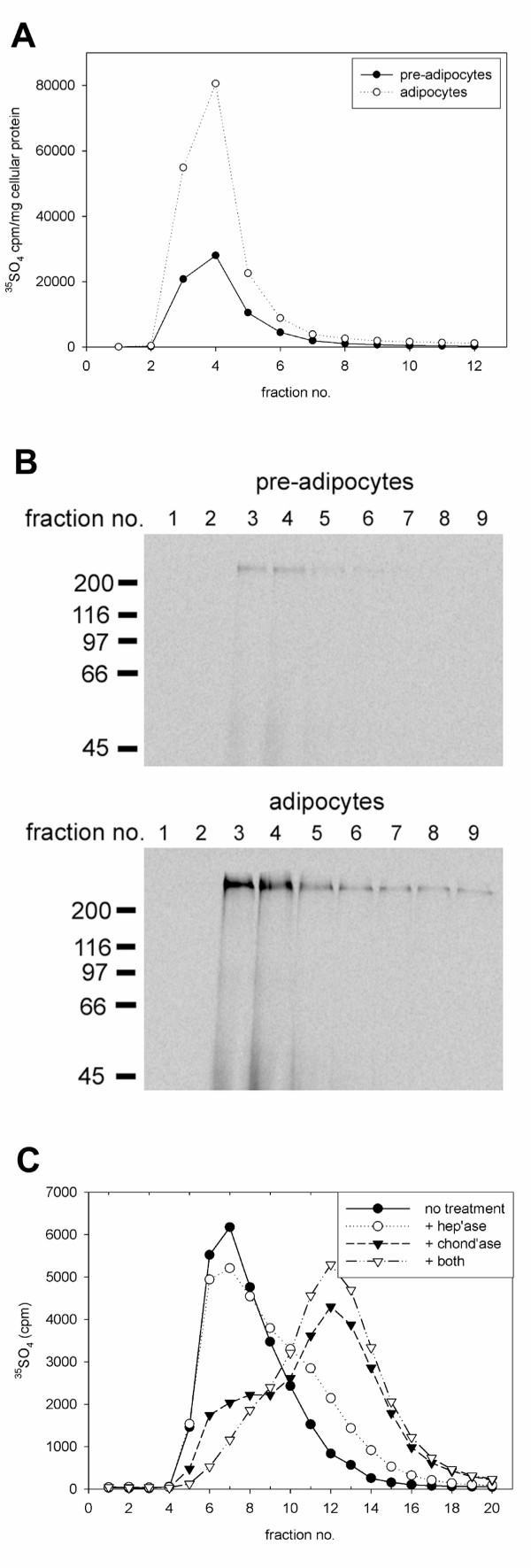
**Adipocyte differentiation results in increased synthesis of a high molecular weight proteoglycan that consists of a mixture of heparan and chondroitin sulfate glycosaminoglycan moieties. **Pre-adipocyte 3T3-L1 cells and differentiated adipocytes were incubated for 20 h with ^35^SO_4 _(125 μCi/ml). (A), cells were lysed with 8 M urea and proteins were fractionated by anion exchange chromatography as described in Methods. Radioactivity in each fraction was determined by scintillation counting. (B), fractions from (A) were separated by 7% SDS-PAGE and subjected to phosphorimager analysis (upper panel, pre-adipocytes; lower panel, adipocytes). (C), ^35^SO_4_-labeled proteoglycans, enriched by anion exchange chromatography, were incubated with or without heparinase I (5 Units/ml), chondroitinase ABC (5 Units/ml), or both enzymes for 20 h at 37°C. Labeled material was then fractionated by size exclusion chromatography and fractions were assessed by scintillation counting.

Although HSPG are known to play a critical role in binding of lipoproteins to the cell surface [46,47] and additionally serve as a primary interaction site for LPL [48,49], little evidence has been reported as to their function in lipid accumulation by adipocytes. To begin to characterize their role in adipocyte growth, we first examined changes in overall proteoglycan synthesis during adipocyte differentiation. 3T3-L1 preadipocytes and differentiated adipocytes were incubated with ^35^SO_4 _to label glycosaminoglycan moieties. Cell lysates were prepared by extracting with urea and radiolabeled proteoglycans were analyzed by anion exchange chromatography. As shown in Fig. 3A, the amount of ^35^SO_4_-labeled proteoglycan recovered from mature adipocytes was ~2.5-fold greater than that extracted from pre-adipocytes indicating that proteoglycan synthesis is augmented during differentiation. When the chromatographic fractions were separated by SDS-PAGE, interestingly, we found primarily high molecular weight species of proteoglycans in extracts from either pre-adipocytes or mature adipocytes (Fig. 3B).

To analyze the composition of the high molecular weight proteoglycan species synthesized by mature adipocytes, we incubated the chromatographically enriched material with heparinase I, chondroitinase ABC, or a mixture of both enzymes followed by size fractionation (Fig. 3C). With no enzymatic digestion, the material eluted at the exclusion limit of the column. Treatment with heparinase I reduced peak fractions 6–8 by ~15% and yielded a lower molecular weight product that eluted between fractions 10–14. By contrast, chondroitinase ABC treatment reduced the high molecular weight, peak fractions 6–8 by ~65–70% and generated mostly low molecular weight products eluting between fractions 11–15. The high molecular weight material remaining after chondroitinase ABC treatment likely consists primarily of heparan sulfate moieties. This was confirmed by treating the enriched proteoglycans with both heparinase I and chondroitinase ABC which resulted in essentially a quantitative shift of labeled material from high molecular weight fractions to fractions consisting of primarily lower molecular weight products. These data provide us with two important observations; 1) major proteoglycan species synthesized by mature adipocytes are of very high molecular weight, and 2) this proteoglycan structure consists mostly of chondroitin sulfate glycosaminoglycans with a smaller percentage of heparan sulfate moieties.

### HSPG participate in the uptake of DiI-labeled apolipoprotein E-enriched VLDL by adipocytes

**Figure 4:**
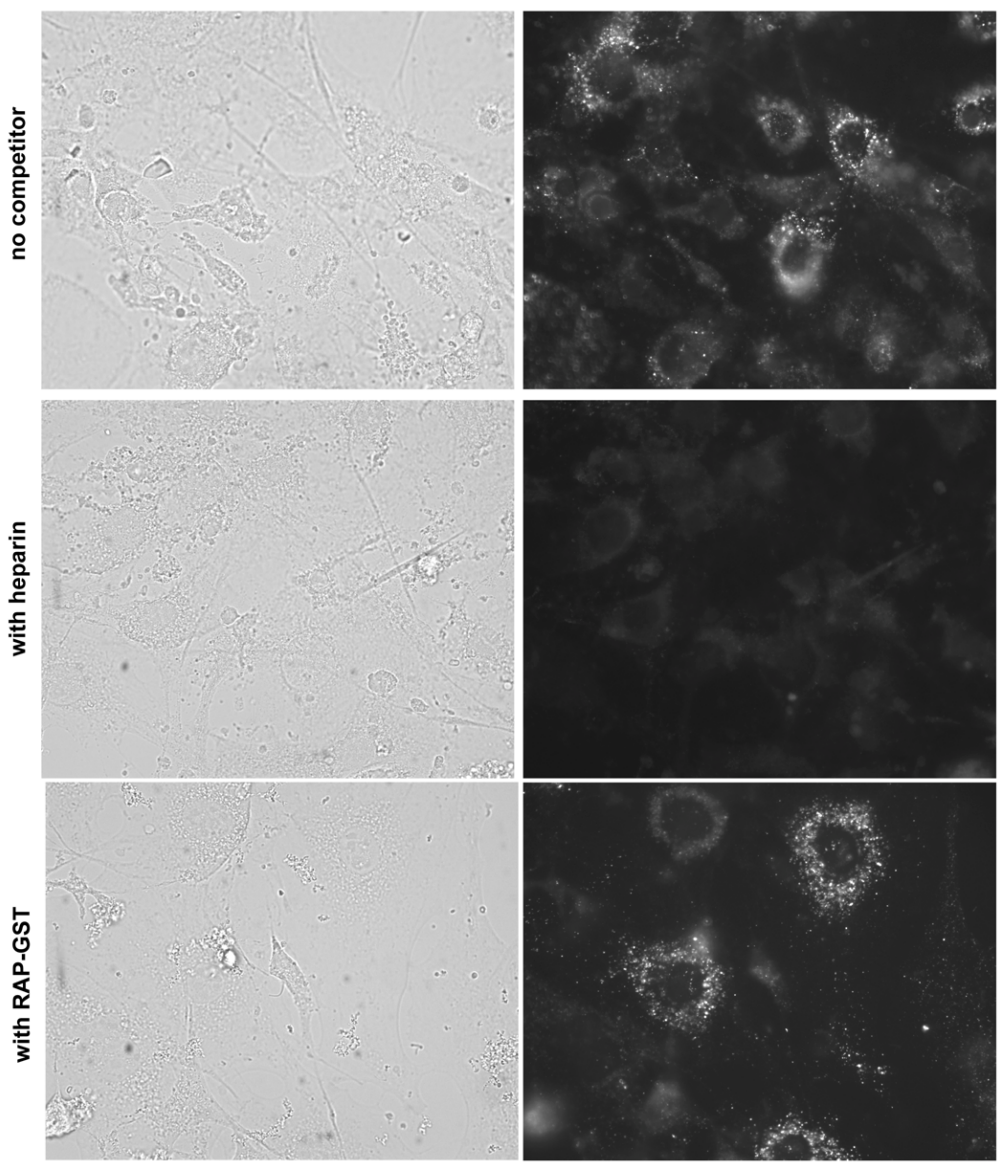
**Heparin, but not RAP, competes for DiI-apoE-VLDL uptake by adipocytes. **Differentiated adipocytes were cultured on glass coverslips and incubated with DiI-labeled apoE-VLDL (4 μg/ml) in the presence or absence (upper panel) of either heparin (500 μg/ml, middle panel) or RAP-GST (50 μg/ml, lower panel) at 37°C for 3 h. Cells were then fixed and processed for fluorescence microscopy. Left panels, phase contrast image; right panels, rhodamine filter set (550 nm excitation-573 nm emission). Magnification, 630×.

The extent to which extracellular lipoproteins contribute to lipid accumulation by adipocytes has not been fully established. A recent study suggests that uptake of VLDL by adipocytes stimulates intracellular lipid accumulation [50]. To identify if either HSPG or LDL receptor family members play a role in this process, we incubated mature adipocytes with DiI-labeled apoE-enriched VLDL in the presence or absence of either heparin (to inhibit HSPG activity) or RAP (to inhibit VLDL-R and LRP function) and visualized the cells by fluorescence microscopy (Fig. 4). In the absence of potential competitors, mature adipocytes readily internalized DiI-apoE-VLDL into small cytosolic vesicles (upper panel). Incubation with RAP showed little or no effect on DiI-apoE-VLDL uptake (lower panel). However, incubation with heparin significantly inhibited intracellular accumulation of DiI-apoE-VLDL (middle panel) indicating that HSPG, rather than VLDL-R or LRP, play a major role in apoE-VLDL internalization.

### HSPG are necessary for intracellular lipid accumulation in mature adipocytes

**Figure 5:**
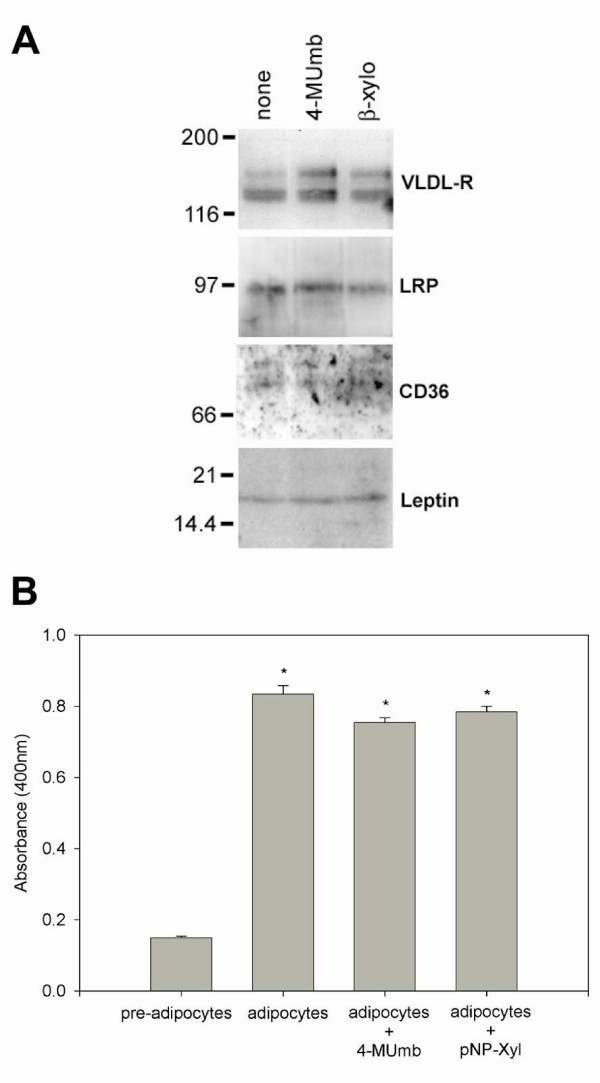
**Inhibitors of HSPG maturation have no effect on adipocyte differentiation. **3T3-L1 pre-adipocytes were incubated with or without 3 mM pNP-Xyl or 4-MUmb concurrently with differentiation reagents for 4 d. Media was then changed to include just insulin with or without 3 mM pNP-Xyl or 4-MUmb for an additional 4–8 d. (A), cell lysates were immunoblotted with anti-VLDL-R mAb (1:50, culture supernatant), anti-LRP antisera (1:2000), or anti-CD36 IgG (5 μg/ml). For anti-leptin, proteins in culture supernatant were concentrated 10-fold by acetone precipitation prior to SDS-PAGE fractionation and immunoblotted with anti-leptin IgG (2 μg/ml). (B), following differentiation and xyloside treatment, cells were cultured for 16 h in serum-free, phenol red-free media. Culture supernatants were then assayed for LPL enzymatic activity as described in Methods. No significant difference was found in LPL levels between adipocytes treated with xylosides and untreated adipocytes (asterisk, p-value <0.01).

4-methylumbelliferyl-β-D-xylopyranoside (4-MUmb) and p-nitrophenyl-β-D-xylopyranoside (pNP-Xyl) are reagents that can serve as alternative acceptors within cells for heparan sulfate moieties and are thus able to compete for heparan sulfate chain addition to proteoglycan core proteins [51,52]. The bare proteoglycan core proteins that result from this treatment traverse to the plasma membrane but are devoid of any heparan sulfate glycosaminoglycan modifications. This in effect removes heparan sulfate proteoglycan functionality from the surface of treated cells and permits an examination of HSPG contributions to cell function. To ensure that these reagents do not interfere with the events of adipocyte differentiation, we treated 3T3-L1 pre-adipocytes with 4-MUmb or pNP-Xyl concurrently with the addition of differentiation reagents and examined the expression levels of proteins known to increase during adipocyte formation, namely VLDL-R, LRP, CD36/FAT [21], and leptin [53]. By immunoblot analysis, we found that the expression levels of these proteins were comparable between treated and non-treated cells (Fig. 5A). Since lipoprotein lipase (LPL) synthesis is also known to increase following 3T3-L1 differentiation [54,55], we measured enzymatic activity of LPL in the culture supernatants of induced and non-induced 3T3-L1 cells and compared these results with those obtained from induced 3T3-L1 cells treated with either 4-MUmb or pNP-Xyl (Fig. 5B). As expected, conversion of 3T3-L1 pre-adipocytes to mature adipocytes resulted in ~5–6-fold increase in LPL activity. Importantly, no significant difference in LPL activity was noted between normal mature adipocytes and those treated with either 4-MUmb or pNP-Xyl. These data indicate that expression of these proteins is unaffected by inhibited proteoglycan maturation and, importantly for our purposes, that addition of 4-MUmb or pNP-Xyl to cells does not prevent adipocyte differentiation.

**Figure 6:**
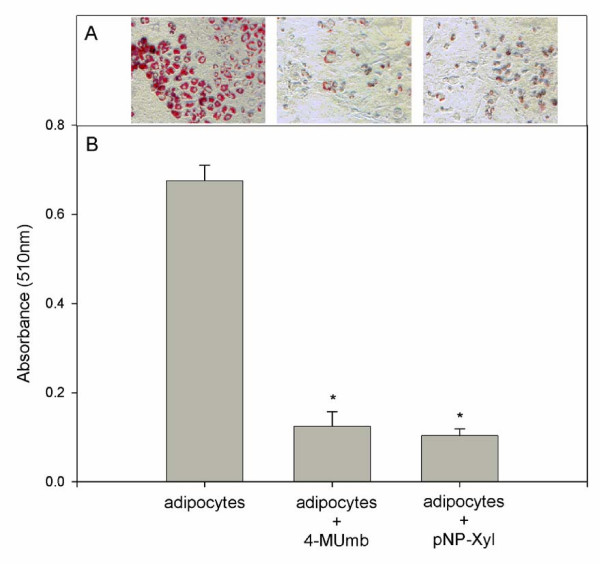
**Reduction of cell surface heparan sulfate glycosaminoglycans significantly reduces intracellular lipid accumulation in adipocytes. **3 mM 4-MUmb or pNP-Xyl was added to 3T3-L1 cells concurrently with differentiation reagents as in Fig. 5. Cells were then fixed with 10% formaldehyde, stained with 0.1% Oil Red O and photographed with phase contrast optics (A; magnification, 400×). (B), after image capture, cell-associated Oil Red O was extracted with 0.1 N HCl and dye was quantitated by spectrophotometry. Asterisk indicates a statistically significant difference compared to untreated adipocytes (p < 0.01).

**Figure 7:**
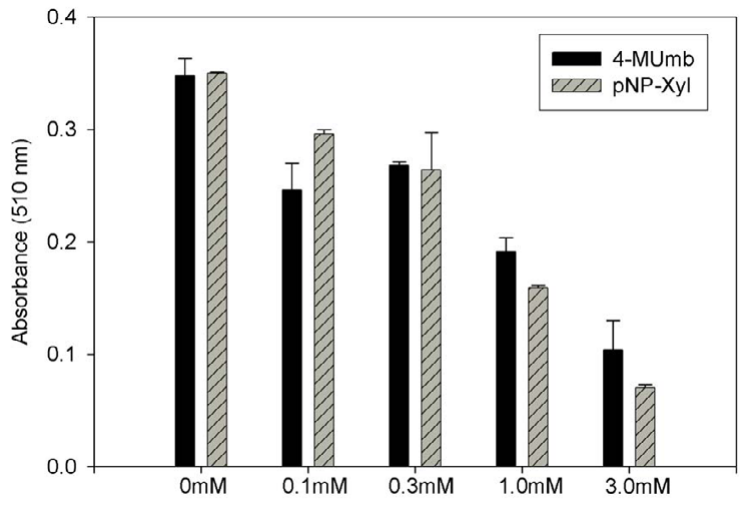
**Reduction in intracellular lipid accumulation in adipocytes by heparan sulfate inhibitors is concentration-dependent. **4-MUmb (solid bars) or pNP-Xyl (hatched bars) was added to 3T3-L1 cells at the indicated concentrations concurrently with differentiation reagents as in Fig. 5. Cells were then fixed with 10% formaldehyde, stained with 0.1% Oil Red O, and cell-associated lipophilic dye was extracted with 0.1 N HCl and quantitated by spectrophotometry. Data shown represents an average of three experiments.

To determine if HSPG are involved in intracellular lipid accumulation by mature adipocytes, we assessed cytosolic lipid droplet formation in induced 3T3-L1 cells following treatment with either 4-MUmb or pNP-Xyl. Again 3T3-L1 pre-adipocytes were incubated with 4-MUmb or pNP-Xyl concurrently with the addition of differentiation reagents and intracellular lipid accumulation was assessed by Oil Red O staining. Upon Oil Red staining, untreated mature adipocytes demonstrated large intracellular droplets; the characteristic morphologic feature of cytosolic lipid accumulation (Fig. 6A, left panel). By contrast, adipocytes treated with either 4-MUmb or pNP-Xyl showed little staining by the lipophilic dye (Fig. 6A, middle and right panels, respectively). To quantitate this effect, cell-associated lipophilic dye was extracted and measured by spectrophotometry (Fig. 6B). Treatment of adipocytes with 4-MUmb or pNP-Xyl resulted in ~6–7-fold decrease in lipid accumulation. Moreover, adipocytes treated with varying concentrations of 4-MUmb or pNP-Xyl demonstrated a concentration-dependent effect of these HSPG inhibitors on intracellular lipid accumulation (Fig. 7). Together, these data indicate that HSPG play an essential role in lipid accumulation by adipocytes.

## Discussion

Intracellular lipid accumulation is a hallmark event of adipocyte development and the major factor in adipocyte hypertrophy. This prompted us to investigate the molecular mechanism by which adipocytes take up extracellular lipid components. In the present study, we provide new information on the expression levels and functional activities of certain cell surface receptors in mature adipocytes that are known to play primary roles in lipoprotein processing and clearance. These receptors have been extensively studied in liver, the major organ for dietary lipoprotein clearance [56], however little information is available regarding their function in lipid accumulation by adipocytes. Using 3T3-L1 cells, which is a well established model of adipocyte differentiation, we show that protein expression of the lipoprotein receptors, VLDL-R and LRP, is increased during adipocyte differentiation, 5-fold and 2-fold, respectively. This increase is consistent with previous studies reporting increases in mRNA levels for these proteins [30,31]. The comparable increase between mRNA and protein levels also suggests that the overall increase in receptor expression following differentiation is primarily due to an increase in transcription with little or no post-transcriptional regulation. We also demonstrate that both pre-adipocytes and mature adipocytes express one major form of sulfated proteoglycan with a relative molecular mass of >500 kD. Expression of this species of proteoglycan increases ~2.5-fold during differentiation and appears to contain both heparan and chondroitin sulfate glycosaminoglycans. Furthermore, we found that uptake of DiI-labeled VLDL by adipocytes is inhibited by heparin, but not RAP, suggesting that in adipocytes HSPG play a greater role in lipoprotein uptake than do members of the LDL receptor family. Since VLDL is a major carrier of fatty acids in the form of triacylglycerols, these data suggest to us that HSPG may provide a mechanism for facilitating the uptake of fatty acids and significantly contribute to intracellular lipid accumulation. To address this hypothesis, we treated adipocytes with 4-methylumbelliferyl-β-D-xylopyranoside and p-nitrophenyl-β-D-xylopyranoside, which are competitive inhibitors of heparan sulfate chain addition, to prevent the synthesis of functional cell surface HSPG molecules. We found this treatment to effectively block intracellular lipid accumulation in adipocytes without affecting adipocyte differentiation.

**Figure 8:**
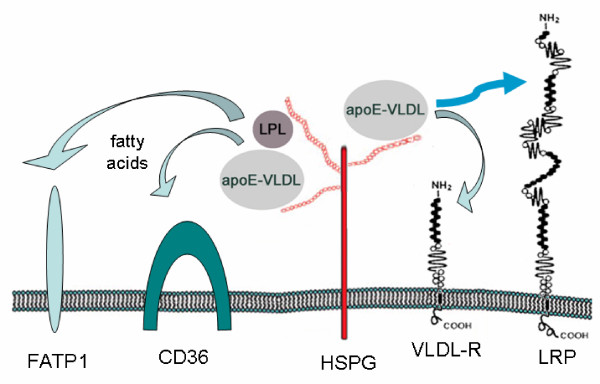
**Model for HSPG activity contributing to fatty acid uptake and intracellular lipid accumulation in adipocytes. **Cell surface heparan sulfate proteoglycans (HSPG) serve as primary binding sites for apoE-enriched VLDL (apoE-VLDL) and lipoprotein lipase (LPL) on adipocytes. Localization of apoE-VLDL and LPL to the cell surface can create a focal reaction center for triacylglycerol hydrolysis thereby releasing fatty acids for cellular uptake by fatty acid transporters such as FATP1 or CD36. Alternatively, similar to that proposed for hepatic clearance of apoE-VLDL and chylomicron remnants [57], initial binding to HSPG serves to concentrate lipoprotein particles at the cell surface and their uptake is mediated either by direct HSPG internalization or following their transfer to VLDL-R or LRP.

These findings offer a novel observation that cell surface HSPG appear to be essential for lipid accumulation and lipid droplet formation in adipocytes. However, this observation also raises the question as to the exact role of HSPG in the transport of fatty acids across the adipocyte plasma membrane. Based on the results presented in this study and those reported elsewhere, we have drawn a testable model to define how HSPG contribute to intracellular lipid accumulation by adipocytes (Fig. 8). HSPG are known to serve as binding sites for apoE-enriched lipoproteins [36-38]. In liver, HSPG are thought to either directly internalize bound lipoproteins by hepatocytes or, alternatively, localize lipoproteins to the cell surface and subsequently transfer particles to LDL receptor family members for endocytosis [57]. In the present study, we show that heparin effectively competes for uptake of DiI-labeled apoE-VLDL while RAP has little or no effect suggesting that, in adipocytes, HSPG are able to internalize apoE-VLDL independent of LDL receptor family members. A direct internalization function by HSPG is also supported by recent findings from our laboratory [47] and others [36,37]. Alternatively, HSPG may serve as a reaction center for triacylglycerol hydrolysis. In addition to apoE-rich lipoproteins, HSPG are also capable of binding LPL [39,40]. The binding function of HSPG for both apoE-rich lipoproteins and LPL can serve to co-localize enzyme and substrate and facilitate release of fatty acids in proximity of the adipocyte cell surface. Uptake of these liberated fatty acids can then be accomplished by fatty acid transport proteins such as CD36 [58] or FATP1 [59].

The identity of this HSPG is currently unknown; however, its large relative molecular mass and composition containing both heparan and chondroitin sulfates are consistent with the structural properties of the syndecan [60,61] and perlecan [62] families of proteoglycans. There are four members to the syndecan family, all of which are type I transmembrane cell surface molecules. Syndecan-1 is found primarily in epithelial and plasma cells, syndecan-2 is found in endothelial cells and fibroblasts, syndecan-3 is expressed in cells of neural crest origin, and syndecan-4 demonstrates a more ubiquitous distribution, including adipose tissue [63]. bFGF treatment increases expression of syndecan-1 in 3T3 cells [64]; however its presence in adipocytes has not been investigated. Although the core proteins of the syndecan family are of modest molecular weight, they are typically modified with the addition of heparan sulfate glycosaminoglycan chains near their N-termini and chondroitin sulfate moieties attached more proximal to their membrane spanning domains, which results in very high molecular masses when resolved by SDS-PAGE, often >500 kD. Perlecan has a large complex modular core protein with a molecular weight of ~400 kD [62,65]. It typically contains three heparan sulfate chains near its N-terminus, but can also have chondroitin sulfate substitutions. Perlecan is a secreted proteoglycan that demonstrates a widespread distribution as a basement membrane component [66]. As both syndecan and perlecan contain heparan sulfate glycosaminoglycan chains, they are able to bind LPL with high affinity [67] and localize its enzymatic activity near the cell surface thereby serving as a reaction center for triacylglycerol hydrolysis. Moreover, studies have shown that they can also bind and internalize lipoprotein particles independent of the classical lipoprotein receptors [37,38]. We are currently in the process of accurately quantitating the amounts of heparan and chondroitin sulfate moieties present on these high molecular weight proteoglycans and identifying their core protein structure. Once this information is available, we will be able to confirm their role in fatty acid transport and adipocyte hypertrophy using expression inhibition procedures and obtain additional information on their role in adipocyte physiology.

The availability of circulating triacylglycerol-rich lipoproteins for adipocyte utilization and storage relies on transport of these particles across the endothelial barrier. Transcytosis of albumin across endothelium has been shown to occur by a vesicular transport pathway [68-70] and is a likely mechanism for transport of fatty acid-albumin complexes. More recently, members of the LDL receptor family, including VLDL-R [71] and megalin [72,73], have also been shown to undergo transcytosis across endothelium and thus provide a mechanism for transport of triacylglycerol-rich lipoproteins into the tissues. Transport of VLDL across the endothelium is also likely to be assisted by hydrostatic and osmotic pressures within the capillary lumen. VLDL and related remnant lipoprotein particles represent the richest source of triacylglycerols in the body. The high metabolic requirements of adipocytes for fatty acids for both storage and utilization make these triacylglycerol-rich particles a suitable source of available fatty acids. The presence of a transendothelial transport mechanism via lipoprotein receptors makes VLDL particles a logical source of fatty acids for adipose growth.

How HSPG might coordinate its activity with CD36 or FATP1 for fatty acid uptake or possibly with members of the LDL receptor family for assisted internalization is under current investigation. Presently, our results provide novel findings indicating that cell surface HSPG activity is necessary for lipid accumulation by adipocytes. It is anticipated that these observations will aid in our understanding of the complex mechanism leading to adipose hypertrophy and obesity, and provide a novel avenue to explore for targeted reduction of intracellular lipid accumulation

## Methods

### Materials

Anti-LRP polyclonal antibody was raised against an 18 amino acid peptide from the cytoplasmic tail of human LRP [74] and anti-RAP polyclonal antibody was raised against a recombinant RAP-GST fusion protein as described [75]. Anti-VLDL-R polyclonal antibody was a generous gift from Dr. Dudley Strickland (School of Medicine, University of Maryland, Baltimore). Anti-VLDL-R monoclonal antibody-producing hybridoma was purchased from American Type Culture Collection (Manassas, VA) anti-leptin polyclonal antibody was purchased from Chemicon (Temecula, CA), and anti-CD36 polyclonal antibody (H-300) was obtained from Santa Cruz Biotechnology (Santa Cruz, CA). Dexamethasone, 3-isobutyl-1-methylxanthine and insulin were obtained from Sigma-Aldrich (St. Louis, MO). ^35^SO_4 _were purchased from MP Biomedicals (Irvine, CA). p-nitrophenyl-β-D-xylopyranoside and 4-methylumbelliferyl-β-D-xylopyranoside were from Calbiochem (La Jolla, CA). Heparin, heparinase I, chondroitinase ABC, p-nitrophenylbutyrate, lipoprotein lipase, and Oil Red O were purchased from Sigma-Aldrich. Optiprep was from Greiner Bio-One (Longwood, FL). DiI (1,1'-dictadecyl-3,3,3',3'-tetramethylindocarbocyanine perchlorate) was purchased from Molecular Probes (Eugene, OR). Apolipoprotein E was obtained from Calbiochem. RAP-GST fusion protein was purified as previously described [76]. Tissue culture plastics were purchased from Corning (Corning, NY) or Greiner Bio-One. Buffers, salts, and detergents were obtained from either Sigma-Aldrich or Calbiochem.

### Cell culture and adipocyte differentiation

3T3-L1 cells were obtained from American Type Culture Collection (Manassas, VA) and grown in Dulbecco's modified Eagle's medium (DMEM) (Invitrogen, Carlsbad, CA) supplemented with 10% (v/v) fetal calf serum (Irvine Scientific, Santa Ana, CA), 1 mM sodium pyruvate, 100 μg/ml streptomycin sulfate, and 100 units/ml penicillin. Cells were cultured at 37°C with 5% CO_2 _and passaged twice weekly. To differentiate 3T3-L1 cells into adipocytes, cells were incubated with 100 ng/ml dexamethasone, 100 μg/ml 3-isobutyl-1-methylxanthine, and 1 μg/ml insulin for 4 days, followed by 1 μg/ml insulin for an additional 4–8 days.

### Immunoblotting

Cell lysates were prepared with 20 mM Tris pH 7.4, 150 mM NaCl (TBS) containing 1% (v/v) Triton-X100. For leptin, culture supernatants were concentrated by mixing with 3 volumes ice cold acetone, incubating at -20°C for 1 h, followed by centrifugation at 10,000 × g at 4°C for 15 min. Protein pellets were resuspended with SDS-PAGE sample buffer supplemented with 2% (v/v) β-mercaptoethanol. Proteins were then separated by SDS-PAGE and transferred to Immobilon-P (Millipore, Billerica, MA) using a wet tank transfer system (BioRad, Hercules, CA). Membranes were blocked with TBS, 0.1% (v/v) Tween-20, 5% (w/v) non-fat dry milk for 20 minutes at 23°C and incubated with the indicated antibody for 2 h at 23°C. Membranes were washed three times (10 min each) with TBS, 0.1% (v/v) Tween-20, and bound antibodies were detected with species-specific HRP-conjugated secondary antibodies (1:3000, BioRad) followed by chemiluminescence detection according to the manufacturer's instructions (Pierce, Rockford, IL). Images were captured using a Syngene GeneGnome system equipped with a Peltier-cooled 16-bit CCD camera and saturation detection. Densitometric analysis was performed using Scion Image, version 4.0.2.

### ^125^I-RAP cell surface 4°C binding assay

RAP-GST was purified [76] and labeled with Na^125^I as previously described [77]. Differentiated 3T3-L1 cells were grown on tissue culture plates precoated with 1% (w/v) gelatin and incubated with ^125^I-RAP-GST (500 ng/ml) diluted into 20 mM Hepes, pH 7.4, 150 mM NaCl, 2 mM CaCl_2_, 1% (w/v) bovine serum albumin (buffer A) at 4°C for 3 h in the presence or absence of a 50-fold molar excess of unlabeled RAP-GST. Unbound ^125^I-RAP-GST was removed by rinsing cells three times with cold buffer A after which cells with bound ligand were solubilized with 0.1 N NaOH. Solubilized proteins were added to EcoLume (ICN Biomedicals, Costa Mesa, CA) and subjected to scintillation counting (Packard Tri-Carb 1600CA, 73% efficiency for ^125^I). Results were normalized to total cellular protein (BCA Protein Assay, Pierce, Rockford, IL). Specificity was determined as the difference between total binding (without competition) and non-specific binding (non-competable) [78]. The actual amount of ligand bound to cells was calculated as cpm ÷ the specific activity of ^125^I-labeled ligand. All data points represent averages of duplicates or triplicates with standard errors of <5%.

### 37°C ligand degradation assay

Differentiated 3T3-L1 cells were incubated at 37°C/5% CO_2 _for 3 h with 500 ng/ml ^125^I-RAP-GST diluted into DMEM containing 1% (w/v) bovine serum albumin in the presence or absence of a 50-fold molar excess of unlabeled RAP-GST. Media was then removed and processed for trichloroacetic acid (TCA) precipitation [75]. TCA-soluble material was added to EcoLume and subjected to scintillation counting. Degradation was calculated as TCA-soluble cpm ÷ specific activity of the radioiodinated ligand.

### ^35^SO_4 _incorporation and proteoglycan analysis

3T3-L1 pre-adipocytes and differentiated adipocytes grown in 100 mm tissue culture dishes were incubated with sulfate-free DME containing 10% (v/v) fetal calf serum and 125 μCi/ml Na_2 _^35^SO_4 _for 20 h at 37°C/5% CO_2_. Cells were detached by incubating with phosphate buffered saline containing 5 mM EDTA for 20 min at 23°C. Following centrifugation to pellet cells (1000 × g, 4°C, 10 min), they were lysed by incubation with solubilization buffer (100 mM Tris, pH 7.5, 150 mM NaCl, 8 M urea) for 1 h at 4°C. Insoluble material was removed by centrifugation (10,000 × g, 4°C, 5 min) and supernatant was batch adsorbed onto 0.5 ml Macro-Prep DEAE Support (BioRad) (pre-washed with solubilization buffer) with gentle agitation for 1 h at 4°C. DEAE resin was applied to a 1.0 cm × 5.0 cm column and washed with 10 column volumes solubilization buffer followed by 10 column volumes 100 mM Tris, pH 7.5, 150 mM NaCl. Bound proteins were eluted with 100 mM Tris, pH 7.5, 1.0 M NaCl and 120 μl fractions were collected. Fifteen μl from each fraction was mixed with 3 ml EcoLume and subjected to scintillation counting. Peak fractions from DEAE enrichment of proteoglycans obtained from labeled differentiated adipocytes (fraction no. 3 and 4, Fig. 3A) were pooled and diluted with dH_2_O to adjust to 150 mM NaCl. This preparation was then incubated with or without either heparinase I (5 units/ml) or chondroitinase ABC (5 units/ml) or both for 20 h at 37°C. The material was applied to a 1.0 cm × 12 cm Sephacryl S-200 HR (Amersham Biosciences, Piscataway, NJ) gel filtration column and eluted with 20 mM Tris, pH 7.4, 150 mM NaCl. 350 μl fractions were collected of which 200 μl was mixed with 3 ml EcoLume and analyzed by scintillation counting.

### Preparation of DiI-labeled apoE-VLDL

New Zealand White rabbits were placed on a high-fat chow diet (10% peanut oil/1% cholesterol) for a minimum of 4 d, blood was drawn into in 1 mM EDTA and centrifuged at 2000 × g, 15 min to remove cells. Chylomicrons were floated by centrifuging plasma at 100,000 × g for 10 min and removed by pipetting. Plasma was then mixed with OptiPrep™ (12% iodixanol final concentration) and centrifuged at 350,000 × g for 3 h (SW55Ti rotor) with slow acceleration and deceleration. VLDL particles (density of 1.006 g/ml) were removed from the top of the gradient by pipetting. Purified VLDL was analyzed by SDS-PAGE and Coomassie R staining to confirm the presence of apoB100 (515 kD) and apoE (35 kD). Animal protocol (#2032) was approved by the University of New Mexico, Health Sciences Center Laboratory Animal Care and Use Committee. For DiI labeling, a working stock of 3 mg/ml was made in dimethylsulfoxide and 0.15 mg was slowly added to 1.67 mg VLDL (in 1.9 ml) with vortexing to rapidly mix. The mixture was then wrapped in foil and incubated for 8 h at 37°C. Unbound DiI was removed from DiI-labeled VLDL by OptiPrep gradient centrifugation as described above.

### DiI-labeled apoE-VLDL uptake assay

3T3-L1 cells were plated on glass coverslips and incubated with differentiation reagents as described above. After conversion to mature adipocytes, cells were rinsed twice with DMEM and incubated with DiI-VLDL (4 μg/ml) and apolipoprotein E (3 μg/ml) diluted into DMEM in the presence or absence of either heparin (500 μg/ml) or RAP-GST (50 μg/ml). After 3 h at 37°C, cells were rinsed with phosphate buffered saline, fixed with 1.5% (w/v) paraformaldehyde for 30 min, and mounted in Gelvatol (Air Products, Allentown, PA) containing 1 mg/ml p-phenylenediamine. Cells were observed with a Zeiss Axioskop microscope equipped for epifluorescence. Images were capture with a Hamamatsu digital/video camera and AxioVision software.

### LPL activity

Treated and non-treated 3T3-L1 cells (as indicated in figure legends) were cultured overnight in serum-free DMEM without phenol red. Media was removed and combined with an equal volume of 100 mM sodium phosphate buffer, pH 7.2, 150 mM NaCl, 0.5% (v/v) Triton-X100. p-nitrophenyl butyrate in acetonitrile was added to a final concentration of 0.5 mM and absorbance at 400 nm was recorded every 10 s for 5 min using a Genesys UV Spectrophotometer. A standard curve for LPL activity was generated by plotting absorbance values obtained with varying concentration of purified LPL (from bovine milk). Quantitation of LPL activity in the individual media samples was determined from the standard curve.

### Oil Red O staining and quantitation

Cells were fixed with 10% (v/v) formaldehyde in phosphate buffered saline for 1 h at 23°C, rinsed twice with water, then stained for 2 h at 23°C with 0.1% (w/v) Oil Red O in 75% (v/v) isopropanol, followed by rinsing twice with water to remove unincorporated dye. Stained cells were viewed and photographed using a Zeiss AxioVert microscope with phase contrast optics and Hamamatsu digital/video camera. For quantitation, cells were dried for 2 h at 37°C, followed by incubation with 100% isopropanol for 15 m at 23°C to extract bound dye. Solubilized dye was then quantitated by spectrophotometry in a BioRad Model 680 Microplate Reader by measuring absorbance at 510 nm. Statistical significance was determined by performing a paired t-test.

## List of abbreviations

ApoE = apolipoprotein E

HSPG = heparan sulfate proteoglycan

VLDL = very low density lipoprotein

VLDL-R = VLDL receptor

LRP = low density lipoprotein receptor-related protein

Syn-1 = Syndecan-1

FATP1 = fatty acid transport protein-1

RAP = receptor associated protein

LPL = lipoprotein lipase

DiI = 1,1'-dictadecyl-3,3,3',3'-tetramethylindocarbocyanine perchlorate

4-MUmb = 4-methylumbelliferyl-β-D-xylopyranoside

pNP-Xyl = p-nitrophenyl-β-D-xylopyranoside

bFGF = basic fibroblast growth factor

## Authors' contributions

LCW carried out the majority of studies and drafted the manuscript. SC performed the xyloside titration study and DN performed the LPL assays. RAO provided the original conceptual framework for the study, carried out pilot experiments, participated in the experimental design and finalized the manuscript for submission. All authors read and approved the final version.
